# Long-duration animal tracking in difficult lighting conditions

**DOI:** 10.1038/srep10432

**Published:** 2015-07-01

**Authors:** Ulrich Stern, Edward Y. Zhu, Ruo He, Chung-Hui Yang

**Affiliations:** 1 Independent researcher, Durham, NC 27705; 2 Dept. of Neurobiology, Duke University, Durham, NC 27710

## Abstract

High-throughput analysis of animal behavior requires software to analyze videos. Such software typically depends on the experiments’ being performed in good lighting conditions, but this ideal is difficult or impossible to achieve for certain classes of experiments. Here, we describe techniques that allow long-duration positional tracking in difficult lighting conditions with strong shadows or recurring “on”/“off” changes in lighting. The latter condition will likely become increasingly common, e.g., for *Drosophila* due to the advent of red-shifted channelrhodopsins. The techniques enabled tracking with good accuracy in three types of experiments with difficult lighting conditions in our lab. Our technique handling shadows relies on single-animal tracking and on shadows’ and flies’ being accurately distinguishable by distance to the center of the arena (or a similar geometric rule); the other techniques should be broadly applicable. We implemented the techniques as extensions of the widely-used tracking software Ctrax; however, they are relatively simple, not specific to *Drosophila*, and could be added to other trackers as well.

Understanding the neural mechanisms that control animal behavior often requires recording animals during behavioral tasks and then analyzing the videos. Human analysis of the videos is both highly labor-intensive and possibly error-prone, making automation very desirable and often critical for achieving acceptable throughput. To address this problem, multiple software systems for analyzing animal behavior have been developed, either for a particular model organism such as *Drosophila*^1^,^2^,^3^,^4^,^5^ or mice^6^,^7^, or less commonly, for multiple species^8^,^9^,^10^. Typically, these systems require the experiments to be performed in good lighting conditions, but this ideal is difficult or impossible to achieve for certain classes of experiments.

Here, we describe techniques that allow long-duration positional tracking in difficult lighting conditions with strong shadows (the term “shadow” includes reflections in this paper) or recurring “on”/“off” changes in lighting. The aspect of long-duration tracking addressed by our techniques is that the “background image” may gradually change. “On”/“off” changes in lighting will likely become increasingly common due to the advent of red-shifted channelrhodopsins (ReaChR^11^,^12^ and Chrimson^13^), as red light penetrates tissue well enough to allow, for example, activation of CNS neurons in adult *Drosophila*, which, in turn, enables studying the effect of activation of different groups of neurons – with precise temporal control – on behavioral output in freely moving adult flies.

The techniques enabled tracking with good accuracy in three types of experiments with difficult lighting conditions in our lab, where we use *Drosophila* egg-laying site selection as model system to study the behavioral and circuit mechanisms underlying simple decision-making processes^14^,^15^,^16^,^17^. First, they enabled tracking for a recent study where we *constantly* illuminated one of the two egg-laying sites with UV light (Fig. 1a,b)^16^, resulting in strong shadows. Second, we used them here to extend some of the results of this study to the case where the site is *periodically* illuminated with UV light, which makes the lighting conditions alternate between Fig. 1b,c. Third, we used the techniques when illuminating the egg-laying chamber with strong red light recurrently to optogenetically activate neurons expressing ReaChR or CsChrimson (Supplementary Figs. S1 and S2).

Instead of writing our own tracking software from scratch, we extended the fly tracking software Ctrax^1^, which is popular for positional tracking of animals both within and beyond the fly community. Our Ctrax extensions (based on Ctrax 0.3.1) are available as project yanglab-ctrax on Google Code (https://code.google.com/p/yanglab-ctrax/). The techniques are relatively simple, not specific to *Drosophila*, and could be added to other trackers as well.

## Results

**Figure 1:**
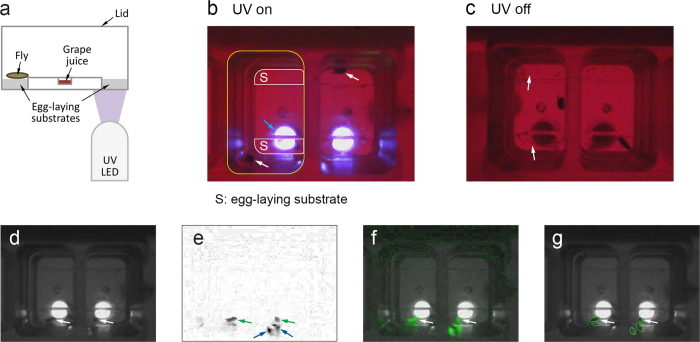
Sample of the difficult lighting conditions our techniques address. (**a**) Schematic of the cross section of our egg-laying chamber. We recorded through the lid with a camera above the chamber. Grape juice provided food for the flies. (**b**) Sample video frame from our “UV on” experiments, showing two chambers. For the left chamber, the top edge of the chamber sidewall is outlined in yellow, and the two egg-laying sites at the bottom of the chamber are outlined in white. The blue arrow points to a UV LED. Note that there is one fly per chamber (white arrows). A red “light pad” under the chambers provides additional lighting – that is invisible to *Drosophila* – for tracking. (**c**) Sample frame from our “UV on/off” experiments at a time when UV is off. The small dark spots on the egg-laying sites are eggs (arrows). (**d-g**) Sample frame with strong shadows that led to false positives, with white and green arrows pointing to the two flies. (**d**) Frame in grayscale, which Ctrax uses for tracking. (**e**) Difference between frame (**d**) and background (see text), with darkness proportional to the absolute value of the difference. The shadows (blue arrows) of the right fly have a larger difference than the fly itself. (We used Ctrax’s “Background Brightness” normalization, which performed best for our chambers.) (**f**) The same difference as in (**e**) now shown in green and superimposed onto the background. (**g**) Flies detected by Ctrax shown as ellipses. The ellipses without arrow are false positives.

We first illustrate how difficult lighting conditions make tracking difficult for an example frame with two flies from our UV constantly on experiments (“UV on”) (Fig. 1d). Tracking software typically detects flies based on the difference between the current frame and the so-called “background,” which is calculated from multiple frames of the video and looks like a frame without the moving objects (flies). The difference between the sample frame (Fig. 1d) and the background is shown in Fig. 1e and, superimposed onto the background, in Fig. 1f. In the difference image (Fig. 1e), some of the shadows of the two flies appear as strongly as the flies, which, in turn, causes the tracking software to mistake shadows for flies, creating false positives (Fig. 1g). For our “UV on” experiments with strong shadows, unmodified Ctrax typically detected hundreds of flies (most of them “short-lived”) in each 8 h video instead of the two flies (one per chamber) we record per video (Table 1).

### Shadow detector

**Figure 2:**
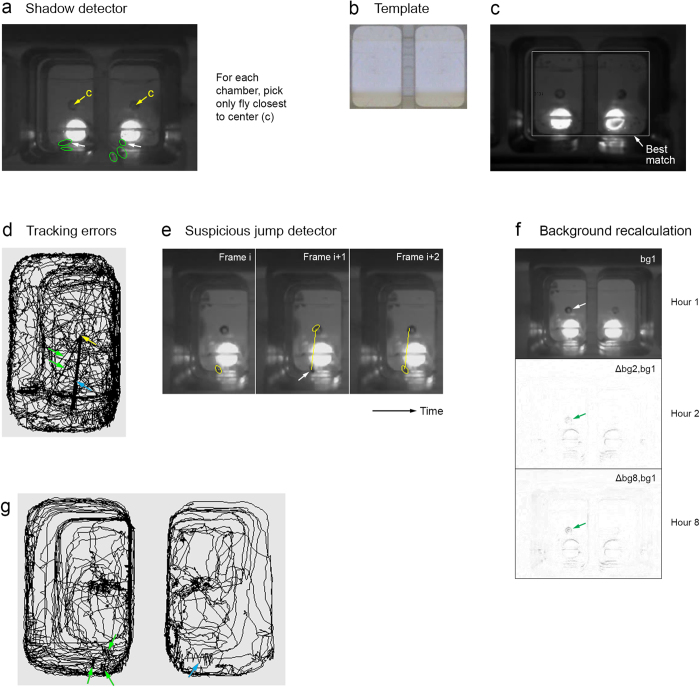
Techniques used for our “UV on” experiments. (**a**) Sample frame (same as Fig. 1g) illustrating shadow detector, which (correctly) picks the flies pointed to by white arrows. See text for details. (**b-c**) Template matching. See text for details. (**b**) Template image (showing bottom of chambers in good lighting conditions). (**c**) Background image with white rectangle indicating where the template best matches the background. (**d-e**) Detecting tracking errors. (**d**) 1-hour trajectory with tracking error. The blue and green arrows point to multiple jumps (straight lines) to or from the center of the chamber (yellow arrow), with the blue arrow pointing to multiple almost identical jumps. (**e**) Sample jump reported by our suspicious jump detector. The image shows three consecutive partial (left chamber only) frames (i, i + 1, i + 2), with the ellipses showing where Ctrax reports the fly is and the arrow pointing to the fly’s actual position in the error frame (i + 1). (**f**) Background recalculation for an 8 h “UV on” video. The background calculated over the 1st hour is shown at the top of the panel (bg1). Differences between bg1 and the backgrounds calculated over the 2nd and 8th hours (Δbg2, bg1 and Δbg8, bg1) are shown below bg1. Some of the background changes here (arrows) are caused by changes in grape juice level in the center well. (**g**) Typical 1-hour trajectories of two (not-too-active) flies using Ctrax with extensions on an 8h “UV on” video with strong shadows. Arrows point to shorter incorrect jumps (easiest to see for blue arrow).

To solve the problem of false positives due to shadows, we extended Ctrax with a shadow detector, which, for each frame, discards all flies but the ones closest to the center of each chamber, taking advantage of the chamber geometry and that we have only one fly per chamber (Fig. 2a).

To calculate the distance to the chamber center for each fly, the shadow detector needs to know the position of the chamber in the frame. We used a total of 18 cameras (Microsoft LifeCams) for recording *Drosophila*, which made the chamber position vary by video. While having the user manually specify chamber centers would have been a viable option, we automated the process instead using template matching, which determines at which position in a large image a small image (“template”) best matches the large image (Fig. 2b,c). We used multiple image transformations (primarily different Canny edge detections) in the matching process and the background instead of a single frame for the large image to avoid flies and their shadows possibly interfering with the matching, achieving high reliability and allowing a template from good lighting conditions (Fig. 2b) to be used also for difficult lighting conditions.

We originally developed the template matching code to determine the positions of the borders of the egg-laying substrates for our behavior analysis (one such analysis is described later in the paper), where the high precision and repeatability template matching provides are more important than in the case of the shadow detector, which would likely have worked well also with approximate positions for the chamber centers.

### Detecting tracking errors

Our shadow detector (Fig. 2a) picks only the detected fly closest to the center for each chamber; if the picked fly is wrong, a tracking error occurs and a jump will appear in the trajectory. In fact, most tracking errors appear as such jumps. But tracking errors can also result in an increase in the number of flies – e.g., due to trajectories’ being broken into pieces – or in flies’ identities being swapped. When using Ctrax with extensions for our tracking tasks, both of these types of tracking errors (i.e., errors without jump) were rare.

To spot possible tracking errors, we hence examined the positional trajectories for “suspicious” jumps for each completed tracking. A trajectory with suspicious jumps is shown in Fig. 2d, where the fly appears to jump to or from the center of the chamber multiple times. The fly did not jump to the center in these cases, however. Instead, the level of grape juice, which we provided the flies with to increase egg-laying (Fig. 1a), in the well in the center of the chamber changed over time, causing false positives that the shadow detector picked over the fly.

To make it less likely that tracking errors are overlooked during manual examination of the trajectories, we also implemented a detector for suspicious jumps (Fig. 2e). The suspicious jump detector reports two types of jumps of at least a certain length: (1) a jump that is followed by another jump in approximately opposite direction and (2) a jump with virtually no movement in the 30s before or after the jump, both uncommon and hence suspicious fly behaviors. We typically reviewed all jumps the detector reported, and in the case of tracking errors, possibly fixed them using Ctrax’s FixErrors tool.

### Background recalculation

Unmodified Ctrax does not handle gradual changes to the background – like the grape juice level change that caused the tracking errors shown in Fig. 2d,e – since it calculates a single background for the whole video. We hence extended Ctrax to recalculate the background typically every 30 or 60 minutes (Fig. 2f). The more frequently the background is recalculated, the better can Ctrax handle gradual changes to the background, but the more likely will a fly that rests for a longer period of time during one of the recalculation periods become part of the corresponding background, causing the fly’s trajectory to be broken into pieces. Such broken trajectories occurred relatively infrequently for us, and if one occurred, it typically had few breaks, making manually fixing it using Ctrax’s FixErrors tool not much effort. Background recalculation every 30 or 60 minutes worked well for us; for some tracking problems with background changes, a more advanced background calculation scheme^18^,^19^,^20^ may be necessary.

### On/off detector

**Figure 3:**
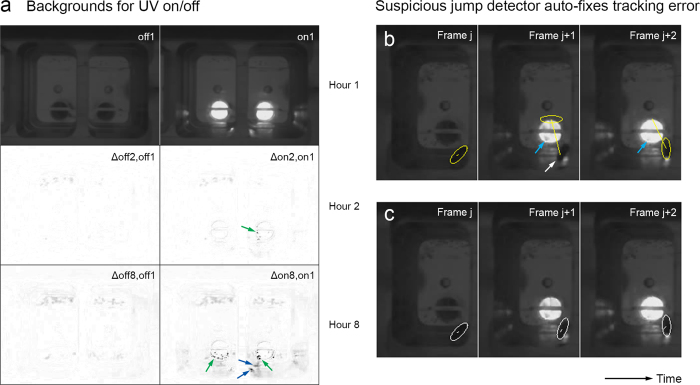
Additional techniques used for our “UV on/off” experiments. (**a**) Backgrounds calculated by the on/off detector for an 8h “UV on/off” video. Separate backgrounds for “on” and “off” were calculated over each hour, with the backgrounds for the 1st hour shown at the top of the panel (off1, on1). Differences between off1 and the backgrounds for “off” calculated over the 2nd and 8th hours (Δoff2,off1 and Δoff8,off1) are shown below off1. Corresponding differences are shown below on1. Note that eggs laid over the UV LEDs caused large differences when the LEDs were on (green arrows), including strong shadows on the chamber sidewalls (blue arrows). (**b-c**) Tracking error fixed by the suspicious jump detector, with the images showing three consecutive partial (left chamber only) frames (j, j + 1, j + 2) both before (**b**) and after (**c**) the fix. The ellipses show where Ctrax reported the fly was and the white arrow points to the actual position of the fly in the error frame. Strictly speaking, not Ctrax but the suspicious jump detector reported the positions in (**c**); the detector is MATLAB code separate from Ctrax. Note that the LED in frame j + 1 is not fully on (compare, e.g., the area of the LED pointed to by the blue arrows in frames j + 1 and j + 2), which caused the suspicious jump.

Unmodified Ctrax cannot handle tracking with recurring changes between two different lighting states (“on”/“off”) since it uses a single background for both states. We hence extended Ctrax with a simple on/off detector. The detector randomly picks 100 frames from the section of the video to calculate the background for, calculates the mean (average brightness) for each frame, and classifies the means into two clusters (“on”/“off”) via k-means. If the cluster centroids differ by more than 3% in brightness, the detector assumes there are two lighting states, a separate background is calculated for each state (Fig. 3a), and the right background is chosen for each frame during tracking. The detector also extends the Ctrax trajectory output file with the information about the timing of the lighting state changes it learned from the video.

The values chosen for the two parameters of the on/off detector – 100 frames and 3% brightness difference – worked well for our “UV on/off” videos. If “on” frames are much more common than “off” frames (or vice versa), more than 100 frames may be required to detect two lighting states. (Assuming that the probability for a frame to be in state “on” is 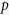 and that the detector picks 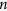 frames independently at random, the probability that the detector is unlucky and chooses all 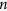 frames from the same lighting state is 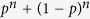; for, say, 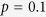 and 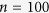, the detector is unlucky just 0.003% of the time.) If the chosen threshold for the brightness difference is too low, the on/off detector can wrongly assume there are two lighting states when there is actually only one; conversely, if it is too high, the detector can wrongly assume there is only one state when there are actually two. To our surprise, even though the 3% threshold was tuned based on “UV on/off” videos, the same threshold also worked well on our strong red light on/off videos that had quite different brightness levels (Supplementary Fig. S1).

### Auto-fixing suspicious jump detector

For our “UV on/off” videos, our suspicious jump detector detected multiple tracking errors that coincided with lighting state changes. The underlying cause of the tracking errors was typically a frame where the lighting level was in-between “on” and “off,” likely due to the UV LED’s changing state during the exposure time of the frame. In case the detector found a suspicious jump that coincided with an on/off state change and was followed by another jump to about the original position, the fly likely performed neither of the two jumps. So we extended the suspicious jump detector to automatically fix such cases (Fig. 3b,c).

### Performance of our techniques for “UV on” and “UV on/off” experiments

Shadow detector and background recalculation enabled tracking 8-hour “UV on” videos with good accuracy (Table 1). Ctrax with extensions correctly detected just two flies for each video, while unmodified Ctrax detected hundreds of flies. We manually examined the 12 jumps our suspicious jump detector reported for the four videos, and tracking was correct in all cases (i.e., the flies *did* jump in these cases). Table 1 does not list “minor” tracking errors that were below the detection threshold of the suspicious jump detector, however. The trajectories regularly had shorter incorrect jumps, usually caused by Ctrax’s regarding fly and shadow as a single object (Fig. 2g). Our behavioral analysis based on the trajectories was essentially unaffected by these minor jumps, allowing us to ignore them.

Combining all the techniques we described enabled tracking 8-hour “UV on/off” videos with good accuracy (Table 2). The correct number of flies was detected for each video. We manually examined all jumps our suspicious jump detector either automatically fixed or just reported. Of the 44 jumps it automatically fixed for the three videos, it made one error but was correct in the remaining 43 cases. So the “auto fix” feature strongly reduced tracking errors here. For the 9 jumps the detector just reported, tracking was correct.

Tables 1 and 2 show the performance of our techniques on representative subsets of our “UV on” and “UV on/off” videos, respectively. (E.g., the performance on our “UV on” videos not shown in Table 1 – we tracked more than 40 “UV on” videos – was similar to the performance on the ones shown in Table 1.) The parameters of our techniques – e.g., for the on/off detector, the number of frames to sample and the threshold for the brightness difference – were manually tuned on just a few of the videos the technique was used for but the chosen parameter values worked well for all of them.

### UV attraction of *Drosophila* virgins in “UV on/off” experiments

**Figure 4:**
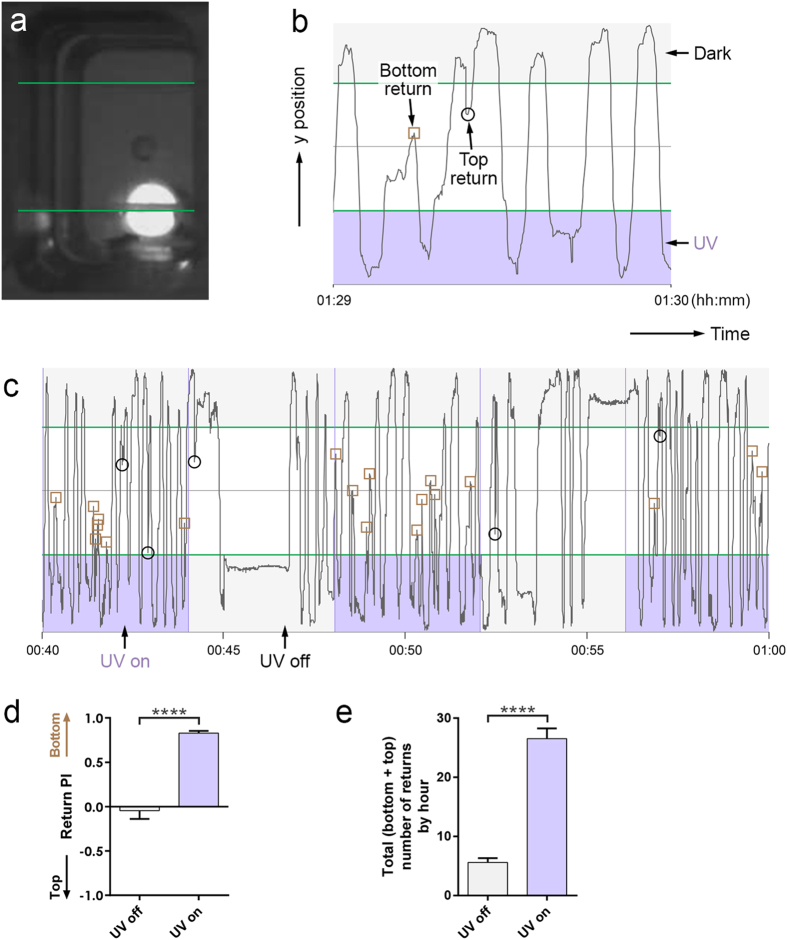
UV attraction of *Drosophila* virgins in “UV on/off” experiments. (**a**) Chamber for “UV on/off” experiments at a time when UV is on. The green lines indicate the borders of the top and bottom sites. (**b**) Plot of y position of a fly in the chamber over 1 minute. A “bottom return” occurs when the fly leaves the bottom site and returns to it without reaching the top site in-between (the brown square marks the point where the fly had gotten the closest to the top site). A “top return” is defined correspondingly. (**c**) Plot of y position of a fly over 20 minutes when the UV on/off state changes every 4 minutes. (**d**) Return preference index (PI) during UV off and UV on times when the on/off state changes every 4 minutes. 12 wild type virgins were recorded for 3 hours each. After tracking, for each hour of the trajectories, we determined the numbers of bottom and top returns for UV off and UV on separately and calculated the return PIs for UV off and UV on as (number of bottom returns - number of top returns) / (total number of returns). n = 34 and 36 hours per bar (two of the hours had no returns for UV off), bars show mean with SEM, unpaired t-test with Welch’s correction, p < 0.0001, two-tailed. (**e**) Total number of returns by hour during UV off and UV on times when the on/off state changes every 4 minutes. n = 36 hours per bar, Mann-Whitney test, p < 0.0001, two-tailed.

We have previously shown that *Drosophila* virgins show strong attraction towards the UV site in our “UV on” experiments^16^. We had measured this attraction by comparing the numbers of “returns” towards the top (dark) and bottom (UV) sites (Fig. 4a,b) using a preference index. For UV constantly on, virgins (and mated females when they are not laying eggs) show a strong preference to return to the UV site.

Since our techniques allow tracking also when one of the sites is *periodically* illuminated with UV, we were curious to also examine this case (Fig. 4c). During times when UV was on, virgins showed strong attraction towards the bottom (UV) site, while there was no such attraction during times when UV was off (Fig. 4c,d). Their attraction for the bottom site hence appears to stop promptly when UV turns off. This result suggests that virgin females’ preference to return towards the UV site is acutely activated by UV illumination as opposed to driven by memory that the site has been illuminated. We also noticed that the virgins seemed to move more when UV is on, which is reflected in a higher total – adding bottom and top – number of returns during times when UV is on compared to when it is off (Fig. 4c,e).

## Discussion

The techniques we described enabled tracking with good accuracy in difficult lighting conditions and over extended periods of time. Our most difficult case (“UV on/off”), which required each of the techniques for good accuracy, combined strong shadows, slowly changing background, and recurring changes between two different lighting states. The techniques enabled us to discover that adult females switch from UV attraction to UV avoidance during periods of high egg-laying^16^, and that their attraction towards UV (when not laying eggs) is activated acutely by UV illumination.

Our shadow detector relies on single-animal tracking – i.e., having a single animal per chamber (or arena) – and on shadows’ and flies’ being accurately distinguishable by distance from the chamber center (or a similar geometric rule). We expect the latter condition to be true for many common arena configurations. Our other techniques – background recalculation, on/off detector, and auto-fixing suspicious jump detector – should be broadly applicable.

While lots of work has been done on “background subtraction”^19^,^20^,^21^ that goes beyond the relatively simple techniques described here, to date even the most advanced techniques have problems with cast shadows, so these techniques are unlikely to enable reliable shadow detection in multiple-animal tracking. But we recently used convolutional neural networks (CNNs)^22^,^23^ – a machine learning technique that leads the object recognition field by a wide margin^24^ – to recognize whether *Drosophila* were “on” (standing or walking) or “off” (not in physical contact with) egg-laying substrates with very low error rate (U.S. and C.-H.Y., in preparation), and CNNs may be able to reliably tell shadows from flies. Similarly, a fingerprinting technique like the one that enabled reliable long-duration tracking of multiple animals in idTracker^10^ may enable reliably telling shadows from flies.

## Methods

This paper primarily describes techniques for tracking in difficult lighting conditions, and we made our implementation of the techniques available as project yanglab-ctrax on Google Code (https://code.google.com/p/yanglab-ctrax/). The UV attraction experiments described extend some of the results we recently published for “UV on” experiments to the “UV on/off” case, and we used the same methods here as we had used then^16^.

## Additional Information

**How to cite this article**: Stern, U. *et al.* Long-duration animal tracking in difficult lighting conditions. *Sci. Rep.* **5**, 10432; doi: 10.1038/srep10432 (2015).

## Supplementary Material

Supplementary Information

## Figures and Tables

**Table 1:** Typical performance of our techniques on “UV on” videos.

sample 8h videos (from UV experiments)		number of flies detected		number of suspicious jumps (Ctrax with extensions)	
video number	UV	unmodified Ctrax	Ctrax with extensions	reported	tracking errors
1	on	216	2	3	0
2	on	285	2	5	0
3	on	183	2	1	0
4	on	262	2	3	0

Results of tracking using unmodified Ctrax and Ctrax with extensions on four “UV on” sample videos (8h each).

**Table 2:** Typical performance of our techniques on “UV on/off” videos.

sample 8 h videos (from UV experiments)			number of suspicious jumps			
video number	UV	number of flies detected	auto-fixed	incorrect fix	reported (unfixed)	tracking errors
5	1 min on/off	2	26	0	2	0
6	2 min on/off	2	14	1	1	0
7	5 min on/off	2	4	0	6	0

Results of tracking using Ctrax with extensions on three “UV on/off” sample videos (8 h each). (We did not run unmodified Ctrax since it was not designed to handle “on”/“off” changes.) “1 min on/off” was 1 min “on,” 1 min “off,” 1 min “on,” etc.
