# Supplementary material for: Long-duration animal tracking in difficult lighting conditions
Source: Sci Rep. 2015 Jul 1;5:10432. doi: 10.1038/srep10432 (PMC4486997; doi:10.1038/srep10432)
Supplement: Supplementary Information [file srep10432-s1.pdf]

Supplementary information

Long-duration animal tracking in difficult lighting conditions

Ulrich Stern, Edward Y. Zhu, Ruo He, and Chung-Hui Yang

Corresponding authors: ulrich.stern@gmail.com and yang@neuro.duke.edu

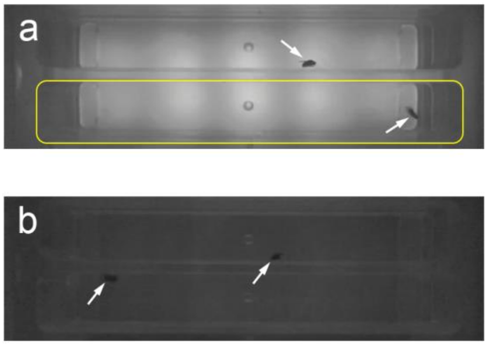

Supplementary Figure S1 | Sample frames from our experiments using strong red light to optogenetically activate neurons expressing ReaChR. (a) Two long chambers wholly illuminated with strong red light. For the bottom chamber, the top edge of the chamber sidewall is outlined in yellow. For both panels (**a**) and (**b**), recording was done in grayscale; in addition, to reduce light intensity for the camera, a filter that lets only blue light (400-500nm) pass (LEE Filters, 713 J.Winter Blue) was placed in front of the camera. Arrows point to flies. **(b)** Same chambers as in (**a**) with red light turned off.

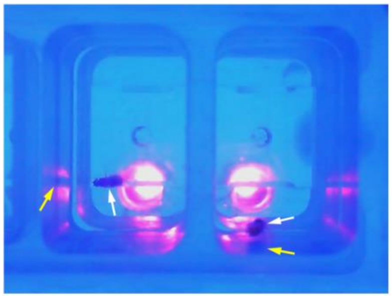

Supplementary Figure S2 | Sample frame from our experiments using strong red light to optogenetically activate neurons expressing CsChrimson. Two chambers partially illuminated with strong red light. White arrows point to flies, yellow arrows to shadows. Like in Supplementary Figure S1, a filter that lets only blue light pass was placed in front of the camera.
